# Distribution of Different PML/RARα bcr Isoforms in Indian Acute Promyelocytic Leukemia (APL) Patients and Clinicohematological Correlation

**DOI:** 10.4084/MJHID.2014.004

**Published:** 2014-01-02

**Authors:** Tathagata Chatterjee, Srishti Gupta, Sanjeevan Sharma, Prosenjit Ganguli

**Affiliations:** 1 Professor & Head of Department, Department of Immunohematology and Transfusion Medicine, Armed Forces Medical College, Pune, India.; 2 Senior Resident, Department of Pathology, NDMC Medical College, Delhi, India.; 3 Associate Professor, Department of Haematology, Army Hospital(R & R, Delhi, India.; 4 Associate Professor, Department of Pathology, Army Hospital(R & R), Delhi, India.

## Abstract

**Context:**

Acute promyelocytic leukemia (APL), an AML subtype, is characterized morphologically by abnormal promyelocytes. Molecular studies show three possible bcr isoforms of PML-RARα fusion gene. This study undertakes analysis of PML-RARα bcr isoforms and their correlation with haematological parameters and response to treatment in Indian patients.

**Aims:**

To study different PML-RARα bcr isoforms in Indian patients and to find any correlation with various haematological parameters and response to treatment

**Settings and Design:**

Patients diagnosed as APL on morphology or flowcytometry and confirmed by RQ PCR were included in the study. Treated APL patients or patients with relapse and on follow-up were excluded from the study.

**Methods and Material:**

Twenty patients over thirty one months period were included. The clinical, haematological & morphological features were analysed, the latter using routine & special cytochemical stains on blood and bone marrow. Flow cytometric evaluation using 4-color Beckman Coulter FC 500 and molecular studies using RT PCR Fusion Quant® kits for bcr-1, bcr-2 and bcr-3 of PML-RARα bcr isoforms on the instrument Rotor Gene™ 3000 were performed.

**Statistical analysis used:**

Student t test was applied to correlate different bcr isoforms with various haematological parameters and response to treatment.

**Results:**

In our study, M:F ratio was 1.5:1 with median age 42 years, Hb - 8.0 g/dl, TLC-7900/μl, and platelet – 35000/μl and varied clinical presentation. Four patients were microgranular variants, and the rest were hypergranular. MPO and CAE positivity were100% and for NSE it was 33.33%. Molecular analysis revealed PML-RARα isoforms of bcr1 in 42.85%, bcr2 in 14.28% and bcr3 in 38.09% patients. No correlation was found between PML-RARα bcr isoforms, different haematological parameters and response to treatment.

**Conclusions:**

Higher incidence of PML-RARα bcr-1 isoform was found in Indian APL patients with no significant correlation between different haematological parameters and response to treatment.

## Introduction

Acute promyelocytic leukemia is a distinct subtype of acute myeloid leukemia (AML), which occurs in 5–13% of patients diagnosed with AML.^1^–^3^ Morphologically the bone marrow shows effacement by heavily granulated cells with folded twisted nuclei. Cytogenetically, it is characterized by balanced reciprocal translocation between chromosome 15 and 17 which results in fusion between promyelocytic leukemia (PML) gene and Retinoic acid receptor α (RARα) gene.^1^

There are 3 possible PML-RARα isoforms caused by these translocations. The breakpoint in chromosome 17 is consistently found in intron 2, but varies in chromosome 15. The 3 breakpoints on the PML gene can occur at intron 3 (L-long form), intron 6 (S-short form), and exon 6 (V form).^4^–^6^ It has been reported that the S (short) form is associated with a shorter remission duration and overall survival compared with the L form in a study by Gonzales et al..^5^ This translocation can be detected by karyotyping or fluorescence in situ hybridization (FISH) studies, and the transcript can be detected by molecular techniques like the polymerase chain reaction (PCR) techniques. The distribution of the breakpoint sites in the PML gene has been reported in several studies from Europe and USA to be approximately 50–55% for PML (L) - RARα, 8–20% for PML (V)-RARα and 27–49% for PML (S)-RARα.^7^ In a study by Dutta et al from India bcr 3 was found to be predominant.^8^

Reports suggesting an association between different PML-RARα bcr breakpoint sites and clinical characteristics or response to treatment in APL patients have not been consistent.^5^–^7^

This study was undertaken to study the predominant PML-RARα bcr isoform in Indian population and to study clinical-haematological profile.

## Subjects and Methods

We analyzed twenty APL patients confirmed by RQ-PCR in the study during the period March 2010 to September 2012. All the patients fulfilled morphologic criteria for APL (Hypergranular and microgranular variant) as classified by WHO classification.^9^ In addition, all patients manifested the PML/RARα rearrangements by RQ PCR. Patients with presumptive morphologic diagnosis but negative molecular result were excluded from this study. Clinical features, hematological parameters [Hemoglobin (Hb), total leukocyte count (TLC), platelet count, prothrombin time and activated partial thromboplastin time, D-dimer and fibrinogen] were analyzed at presentation. Patients were grouped under low risk group (TLC <10,000/μl, platelet >40,000/μl), intermediate risk group (TLC <10,000/μl, platelet count < 40,000/μl) and high risk group (TLC >10,000/μl).^10^

Peripheral blood and bone marrow aspirate were stained with Leishman stain and cytochemistry consisted of myeloperoxidase (MPO), Chloroacetate esterase (CAE) and non-specific esterase (NSE) using Merck’s® Diagnostic reagents and manufacturer specified staining instructions.

Flow cytometric evaluation was done on bone marrow aspirate using Beckman Coulter FC 500 four colour flowcytometer using standard Lyse wash technique. It was done on eleven patients only due to nonavailability of reagents. Antibodies used were labelled with FITC, PE, ECD and PC-5. Antibody panel used was CD13, CD33, CD45, cMPO, cCD79a, cCD3, CD15, HLA-DR, CD10, CD19, CD34, CD7, CD117 and sCD3. Antibody was considered positive if more than 20% of cells gated were positive. Sample was processed within 24 hours of collection. Gating strategy used was CD45 vs. SSC.

Samples were outsourced for cytogenetic evaluation due to lack of facilities in the centre for balanced reciprocal translocation between chromosome 15 and chromosome 17.

For molecular studies, peripheral blood sample was collected. RQ PCR was done for PML-RARα fusion transcript. RNA was extracted and converted to cDNA using Applied Biosystems® High Capacity cDNA Reverse transcription kit on the same day of RNA extraction. cDNA was amplified using Fusion Quant® kits for PML (L)-RARα, PML (V)-RARα and PML(S)-RARα on the instrument Rotor Gene™ 3000 as per the manufacturer’s instructions. ABL transcript was used as an endogenous control gene. Water was used as a negative control. The test samples were run in duplicate.

RQ – PCR was carried out using Taqman® universal PCR Master Mix and IPSOGEN® Primers and probe mix. Three different probes and primers were run for PML (L) - RARα, PML (V)-RARα and PML(S)-RARα all of which had separate kits. The kit used for PMLRARA@ is a quantitative individual primer based one and not multiplexes. Unfortunately, the only kit available as a multiplex one in our country is a qualitative one. The kit used in our dept is tabulated below.

**Table:** 

CODE	QIAGEN Kit NAME
672123	*ipsogen®* PML-RARA bcr1 Kit (24) CE
672213	*ipsogen®* PML-RARA bcr2 Kit (24)
672313	*ipsogen®* PML-RARA bcr3 Kit (24)

The standards of each are labelled as F1, F2, F3, F4, F5. These have 10,100,1000,100,000 and 10,00,000 copies/5ul respectively.

Initially, we ran control gene, in our case ABL gene which has three standards C1, C2, C3 of 1000, 10,000 and 100,000 copies/5ul respectively. If these were in acceptable range then we proceeded for PML(L)-RARα, PML(V)-RARα and PML(S)-RARα separately along with their 5 standards in a single Real time Q PCR and not multiplex.

Total number of cycles run was 45 cycles over a period of 2 hours and 20 minutes. Standard curves were obtained for control gene, fusion gene of standard and test samples after acquisition

The Control Gene (CG) standard curve equation was used to transform raw cycle threshold (Ct) values obtained with Primer & Probe Mix for Control gene (CG-PPC) for the unknown samples, into the CG copy numbers (CG CN).

The Fusion Gene standard curve equation was used to transform raw Ct values (obtained with Fusion gene Primer & Probe Mix (FG-PPF) for the unknown samples into Fusion Gene copy numbers (FG CN). The ratio of these copy numbers gives the normalized copy number (NCN):

NCN=FG CN/CG CN

Any value of normalized copy number above zero was considered as significant.

Patients were treated with all Trans retinoic acid (ATRA) based regimen. Consolidation strategies adopted in our Institute depended on the risk classification for relapse at diagnosis. Two or 3 cycles of anthracycline-based chemotherapy were administered for low- and intermediate-risk patients in first complete remission (CR1) as shown in table 1. For patients with high-risk disease (WBC>10 000/μL), either intermediate- or high-dose Ara-C was administered depending on age as a first consolidation as done in the GIMEMA APL 2000 study or ATO for 2 courses of 25 days each as carried out in the second North American Intergroup C9710 study.

## Results

Twenty patients were studied. Patient characteristics are given in table 2. The presenting clinical features are given in table 3.

On morphological analysis, four patients were microgranular variants and sixteen were hypergranular. MPO and CAE stains were seen positive in 100% of APL cases, and NSE was seen in 33.33% of cases. Flowcytometric analysis of eleven patients [microgranular-n (2), hypergranular-n (9)] revealed characteristic findings of APL [11]. All hypergranular variants revealed a high side scatter and antigenic expression of CD13, CD33 and MPO. CD117 was expressed in one hypogranular and two hypergranular variants. Weak CD34 expression was seen in two hypogranular variants. HLA-DR expression was not seen in any case. Weak CD15 was noted in three hypergranular variants.

All the patients were cytogenetically determined to be characteristic t (15; 17) (q22; q21).

Molecular analysis revealed PML(L) RARα in 42.85%, PML(V) RARα in 14.28% and PML(S) RARα in 38.09% patients. Amongst these, two microgranular variants were PML(L) RARα positive, third microgranular variant was PML(V) RARα and the fourth PML(S) RARα positive. Five patients died during the induction phase of which three were PML(S) RARα positive and two were PML(L) RARα positive. Three patients died due to intracranial haemorrhage, and two patients died due to hyperkalemia leading to cardiac arrest. Rest of the patients achieved molecular remission. No significant correlation was found between PML-RARα bcr isoforms, age, sex and different haematological parameters namely haemoglobin, total leukocyte count, platelet count and response to treatment.

## Discussion

On examination, one unusual feature was gum hypertrophy seen in four patients (20%). This observation is similar to a previous Indian study by Dutta et al.^8^ where the same clinical feature was observed in some of their patients. However unlike that study not a single case of ours presented with scrotal ulceration. We feel that presence of gum hypertrophy seen commonly in acute myelomonocytic leukemia is also seen in Indian APL patients. Generally, gum hypertrophy is very rarely seen in AML-M3.^12^ Another unusual feature was high TLC at presentation. TLC varied from 3000/μl to 71,000/μl with a median of 7900/μl. APL is characterised by pancytopenia in peripheral blood. Raised TLC is seen in 10–30% of cases especially with microgranular variant.^12^ In our series of twenty patients, four were microgranular variants (20%). Three out of four patients of microgranular variants showed high TLC at presentation. Statistical significance was not determined due to the small number of cases.

Cytochemistry results showed positivity for MPO and CAE in all the APL cases (100%). Interestingly 33.33% of cases also showed positivity for NSE. This finding is in concordance with previous published literature where NSE positivity in APL cases is between 13.5% – 60.7%.^8^,^13^

RQ-PCR done at the time of diagnosis revealed PML-RARα transcript in twenty patients. Unusual feature was that in our study PML(L) RARα isoform was found to be the predominant isoform (42.85%) followed by PML(S) RARα isoform (38.09%). Whereas according to data published in India by Dutta et al.^8^ PML(S) RARα isoform was found to be significantly high (72.7%). However, our study results were in concordance with published western studies.^7^ In a study of 2003 Douer et al.^7^ found a frequency of PML(L) RARα isoform significantly high. They summarized also the distribution of the breakpoint sites in the PML gene reported in several studies from Europe and the USA, which was approximately 50–55% for PML(L) RARα, 8–20% for PML(V) RARα and 27–49% for PML(S) RARα.^7^ This finding suggests that PML (L) RARα subtype may represent a distinct biological subset and breakpoint at intron 6 in PML gene may not be a random event. This might be possibly related to genetic and/or environmental factor(s) playing a role in determining the breakage site of the PML gene. Multicentre trial studies are needed to confirm the same.

Of total twenty patients in the study, five patients (25%) died during the induction phase. Three were PML(S) RARα positive and two were PML(L) RARα positive. No mortality was noted with PML(V) RARα isoform. In previous studies of cytotoxic chemotherapy, early deaths during induction in patients with APL occurred primarily as a consequence of intracranial hemorrhage.^14^,^15^ The hemorrhagic diathesis of APL is related to depletion of platelets and clotting factors, probably owing to leukemic cell lysis and release of procoagulant or fibrinolytic materials into the circulation.^16^,^17^ Early mortality from this problem has ranged from 10% to 47% in published series.^18^,^19^ According to published studies PML(S) RARα isoform has been related to inferior duration of remission and overall survival.^20^

All our 15 patients are under regular follow-up. There is a system in the Indian Armed Forces patients to compulsorily attend follow-up as per standard instructions of the Clinicians. A separate long-term follow-up study is being devised by our Clinical investigators for all APL patients and is likely to be published in two years time involving at least 50 patients; hence it will be beyond the scope of this particular publication to consider the over-all DFS, median FU and OS. However, regular follow-up is being meticulously recorded for all our fifteen APL patients.

No significant correlation was found between the PML-RARα isoforms, haematological parameters, age, sex and response to treatment; however study involving a small sample size may not have the power to detect such a relationship. This is similar to results of previous studies.^7^,^21^ However some studies have found an association between PML(S) RARα isoform and high TLC^22^ and PML(V) RARα isoform and high TLC at presentation.^5^

## Conclusion

To conclude, APL is a distinct biological entity. In our study PML(L) RARα isoform was found to be the predominant isoform (in concordance with western studies) followed by PML(S) RARα isoform with no significant correlation with age, sex, haematological parameters and response to treatment.

## Figures and Tables

**Table 1:** 

Induction	ATRA – 45 mg/m^2^ oral daily till maximum 60 days/haematological remission Injection Idarubicin 12mg/m^2^ once daily (D-1 to D-3) concomitantly
Consolidation (2–3 cycles depending upon risk stratification)	Injection Idarubicin 12mg/m^2^ daily for 3 days ATRA 45mg/m^2^ once daily for 15 days
Maintenance	3 monthly cycles × 6 cycles 3 monthly cycle- Day 1- 15:ATRA 45 mg/m^2^ in two divided doses Tab 6- Mercaptopurine 90mg/m^2^ 3 weeks/month Tab Methotrexate 15mg/m^2^ once a week × 3 weeks/month

**Table 2:** Patient Characteristics

No. of patients	20
M:F ratio	1.5:1
Median age	42 years(Range 14 – 62 years)
Median Hemoglobin	8.0 g/dl (Range 4.5 –10.8 g/dl)
Median Total leukocyte count	7900/μl (Range 3000 – 71000/μl)
Median platelet count	35000/μl (Range 10,000 – 52,000/μl)
Median PT	17.4 seconds (Range 14.2 – 23 seconds) (PT control - 13 seconds)
Median Aptt	40.1 seconds (Range 34.1 – 47 seconds) (aPTT control - 40.1 seconds)
Median D-dimer	5.3 μg/ml (Range 0.8 – 8.1 μg/ml) (Normal value < 0.5)
Median fibrinogen	98 mg/dl (Range 80 – 312 mg/dl ) Normal value 100 – 400 mg/dl)
Low risk group	2 patients
Intermediate risk group	9 patients
High risk group	9 patients

**Table 3:** 

Isoforms	Total patients(n)	Pallor	Fever	Loss of appetite	Bleeding manifestationsn	Gum hypertrophy	DIC
PML(L)-RARα	9	100%(9)	55.55%(5)	33.33%(3)	44.44%(4)	Nil(0)	44.44%(4)
PML(V)-RARα	3	100%(3)	100%(3)	Nil(0)	100%(3)	33.33%(1)	66.66%(2)
PML(S)-RARα	8	100%(8)	100%(8)	62.5%(5)	62.50%(5)	37.50%(3)	62.50%(5)
